# “Long COVID” results after hospitalization for SARS-CoV-2 infection

**DOI:** 10.1038/s41598-022-13077-5

**Published:** 2022-06-10

**Authors:** Marta Rigoni, Emanuele Torri, Giandomenico Nollo, Livia Delle Donne, Sebastiano Rizzardo, Lorenza Lenzi, Andrea Falzone, Susanna Cozzio

**Affiliations:** 1 BIOtech Laboratories, Department of Industrial Engineering, University of Trento, Via Sommarive 9, Povo, 38123 Trento, Italy; 2 Department of Biomedical, Surgical and Dental Sciences, University Statale of Milan, Milan, Italy; 3 Dipartimento Salute e Politiche Sociali, Provincia Autonoma di Trento, Trento, Italy; 4 U.O. di Medicina Interna, Ospedale di Rovereto, Azienda Sanitaria per i Servizi Provinciali di Trento, Trento, Italy; 5 Unità Operativa Multizonale di Radiologia Ospedale di Rovereto e Arco, Azienda Sanitaria per i Servizi Provinciali di Trento, Trento, Italy

**Keywords:** Diseases, Health care, Medical research, Risk factors, Signs and symptoms

## Abstract

Long-term sequelae of symptomatic infection caused by SARS-CoV-2 are largely undiscovered. We performed a prospective cohort study on consecutively hospitalized Sars-CoV-2 patients (March–May 2020) for evaluating COVID-19 outcomes at 6 and 12 months. After hospital discharge, patients were addressed to two follow-up pathways based on respiratory support needed during hospitalization. Outcomes were assessed by telephone consultation or ambulatory visit. Among 471 patients, 80.9% received no respiratory support during hospitalization; 19.1% received non-invasive ventilation (NIV) or invasive mechanical ventilation (IMV). 58 patients died during hospitalization, therefore 413 were enrolled for follow-up. At 6 months, among 355 patients, the 30.3% had any symptoms, 18.0% dyspnea, 6.2% neurological symptoms. Fifty-two out of 105 had major damages in interstitial computed tomography images. NIV/IMV patients had higher probability to suffer of symptoms (aOR = 4.00, 95%CI:1.99–8.05), dyspnea (aOR = 2.80, 95%CI:1.28–6.16), neurological symptoms (aOR = 9.72, 95%CI:2.78–34.00). At 12 months, among 344, the 25.3% suffered on any symptoms, 12.2% dyspnea, 10.1% neurological symptoms. Severe interstitial lesions were present in 37 out of 47 investigated patients. NIV/IMV patients in respect to no respiratory support, had higher probability of experiencing symptoms (aOR = 3.66, 95%CI:1.73–7.74), neurological symptoms (aOR = 8.96, 95%CI:3.22–24.90). COVID-19 patients showed prolonged sequelae up to 12 months, highlighting the need of follow-up pathways for post-COVID-19 syndrome.

## Introduction

Coronavirus disease 2019 (COVID-19) is a severe acute respiratory infection caused by the emergent coronavirus, SARS-CoV-2. Though most infected individuals are asymptomatic, SARS-CoV-2 infection may cause symptoms ranging from mild to severe acute respiratory distress, with a substantial fraction of patients requiring hospitalization (estimated to be around 10%) and many patients experiencing prolonged symptoms and complications for weeks or months after the initial period of acute illness^1–6^.

The long COVID-19 phase or post-acute sequelae (signs and symptoms that continue or develop after acute COVID-19) includes both ongoing symptomatic COVID-19 (from 4 to 12 weeks) and post-COVID-19 syndrome (12 weeks or more)^6,7^ and are not explained by an alternative diagnosis. Therefore, patients should be followed up to detect and manage sequelae and functional impairment^8,9^.

Since COVID-19 has spread globally, and long COVID became a burgeoning health concern^10^, a growing number of studies has been focused on “long-term effects of COVID-19”^11–18^. However, to date only a few studies^19–24^ really addressed long term (up to 12 months) sequelae for hospitalized patient populations, as well as temporal trends and long-standing health consequences for “long-haulers” with a whole patient pathway perspective in different populations, timepoints, settings and countries. The full range of long-term health consequences of COVID-19 in patients who were discharged from hospital is largely unclear^25^.

We implemented a stratified follow-up pathway and performed a prospective observational cohort study in patients who survived hospitalization for acute COVID-19 with the aim to:

- investigate the persistence of COVID-related symptoms and long-term sequelae at six and twelve months after hospital discharge;
- assess whether patients who received more robust respiratory support during their hospitalization experienced more long-term specific effects of COVID-19.

## Methods

### Study design and participants

We conducted a prospective observational cohort study in adult patients with COVID-19 who had been admitted to the Internal Medicine ward of the Santa Maria del Carmine Hospital of Rovereto (Italy), between March 1, 2020, and May 31, 2020. The setting was a public general hospital with 300 beds that was identified in the first phase of the Italian COVID-19 outbreak as a regional hub hospital for COVID patients in the Autonomous Province of Trento (northeastern Italy, around 543,000 inhabitants).

All COVID diagnoses were confirmed using molecular diagnostic tests for SARS-CoV-2 (Reverse Transcription Polymerase Chain Reaction, RT-PCR) performed with a nasopharyngeal swab, oropharyngeal swab, or bronchoalveolar lavage using the standard protocols determined by the Italian Health Ministry, consistent with the World Health Organization’s interim guidance diagnostic criteria for adults with severe COVID-19 pneumonia^26,27^.

We set up a long-term stratified follow-up pathway supported by structured and recorded phone call that enabled patient monitoring and evaluation and the recording of long-lasting symptoms and clinical findings in patients hospitalized with COVID-19.

We implemented two different follow-up routes targeting two different patient groups on the basis of the severity of COVID disease assessed during hospitalization through the Brescia-COVID Respiratory Severity Scale (BCRSS), an algorithm developed in Italy during the early phase of the COVID-19 pandemic to aid the assessment and management of patients with COVID-19 pneumonia^28^. The score was calculated for each patient at the time of admission and in the case of clinical worsening during hospital stay; for patient stratification, we used the worst score recorded.

We further categorized and routed patients as follows:

- cohort one: patients with mild COVID-19, treated without oxygen or with nasal cannula or mask to administer supplemental low flow oxygen-therapy < 6 l/min (BCRSS 0–1). This group was given a phone follow-up;
- cohort two: patients with moderate to severe disease, requiring oxygen-therapy > 6 l/min, High-Flow Nasal Cannula (HFNC), Non-Invasive Ventilation (NIV) including Continuous Positive Airway Pressure (CPAP), or invasive mechanical ventilation (IMV) (BCRSS ≥ 2). All patients in IMV previously failed to recover on HFNC or NIV. This group was given an ambulatory follow-up visit (in-person).

Shifting between cohorts:

- regardless of the severity of COVID-19 during hospitalization, patients with significant cognitive impairment or motor disabilities, residents of long-term care facilities, patients who refused the ambulatory follow up (i.e. because they lived far away from the hospital) were assigned to cohort one (phone follow up);
- patients with a modified British Medical Research Council (mMRC) dyspnea scale ≥ 1 at the time of the phone follow-up were referred to cohort two (ambulatory follow-up).

The time frames for the follow-up visits were as follows: 6 months (25–27 weeks post symptom onset), and 12 months (50–54 weeks post symptom onset).

### Procedures

All patients pertaining to cohort one and two were assessed at 6 months and 12 months after discharge and were investigated for serum levels of SARS-CoV-2 IgG antibodies (6 and 12 months) and status of vaccination anti-SARS-CoV-2 (fully vaccinated individuals) at 12-months, when vaccination was available and indicated for infected individuals according to Italian guidance on vaccination of COVID survivors^29^.

Patients enrolled in route one (cohort one) have been phone interviewed by physicians of the general medicine ward following a structured questionnaire (Table 1S Supplementary material) conceived similarly to other published tools^30^ investigating clinical recovery and presence of COVID related symptoms. Additionally, all patients were assessed with the modified British Medical Research Council (mMRC) dyspnea scale, a five-category scale to characterize the level of dyspnea with physical activity in which higher scores correspond with increased dyspnea (from 0 to 4)^31^. In case of mMRC scale ≥ 1 the patient was shifted to ambulatory follow up at the same timepoint. For people with disabilities or long-term care facilities residents the interview was supported by caregiver or treating physician.

Patients enrolled in route two (cohort two) have been evaluated with follow-up in person. We performed a complete ambulatory visit where patients were interviewed with the same questionnaire used at distance and undertaken the mMRC score. In addition, the same day of the visit, patients performed a functional assessment carried out with Six-Minute-Walking test (6MWT)^32^ which was performed on all patients at 6 months and at 12 months only on patients showing altered 6MWT test at 6 months; as part of the pathway, patients with altered 6MWT were referred to pneumological specialistic consultation to perform additional lung tests. In addition, we performed a chest non-contrast high resolution Computed Tomography (CT) scan on all patients at 6 months and only on survivors with interstitial abnormalities of degree 3 and 4 (see below) at 6 months for the 12-month follow-up.

Main CT patterns, features and distribution of lung abnormalities were described in line with the terms defined by relevant peer-reviewed literature published at the time of the study preparation^33,34^.

The assessed whether lesions identified on the CT involved one or both lungs and several lung lobes. The evaluation of the imaging appearance included: lesion density (ground-glass opacity, consolidation), distribution (unilateral, bilateral), interlobular septa thickening, crazy-paving patterns, bronchiolectasis and bronchiectasis. The degree of chest imaging changes of the lung parenchyma and lung interstitium were graded on a 5-point scale based on the severity of the abnormal finding and the extent of lung involvement.

For parenchymal CT the score was categorized as follows:

- 0 normal;
- 1 unilateral ground-glass opacity (GGO);
- 2 bilateral GGO;
- 3 bilateral GGO and unilateral consolidations;
- 4 bilateral GGO and bilateral consolidations.

For interstitial CT the score was categorized as follows:

- 0 normal;
- 1 unilateral thickening of the subpleural perilobular septa;
- 2 bilateral thickening of the subpleural perilobular septa;
- 3 bilateral thickening of the subpleural perilobular septa and subpleural bronchiolectasis;
- 4 bilateral thickening of the subpleural perilobular septa and subpleural bronchiolectasis and bronchiectasis.

To make statistical analysis easier, CT scores were divided into two categories: normal or mild damage for 0, 1, and 2 scores, and moderate to severe damage for 3, and 4 scores.

All chest CT scans were performed by the hospital radiology unit using a GE Revolution Evo CT scanner (GE Medical System, Boston, Massachusetts, USA). Scanning parameters were tube voltage (100 kV), tube current (10–240 mA), slice thickness (5 mm), interval between slices (5 mm), consecutive 1,25 mm slices for high-resolution reconstruction scan and scanning time (< 5 s). A senior radiologist evaluated the scanned images to identify CT characteristics of each patient. People were placed in a supine position with feet first.

Immunoglobulin G test for SARS-CoV-2 was performed by adopting a fully automated chemiluminescence immunoassay (CLIA) for the quantitative detection of anti‐SARS‐CoV‐2 IgG antibodies^35^. SARS‐CoV‐2 antibodies IgG CLIA kits were from Shenzhen YHLO Biotech Co, Ltd China) with two antigens of SARS‐CoV‐2 coated on the magnetic beads of the CLIA (nucleocapsid protein or N protein and spike protein or S protein). All antibody tests were performed by iFlash1800 CLIA fully automatic analyzer from YHLO biotech Co. The cut-off value proposed by manufacturer for a positive result was 10 AU/mL, samples with values more than or equal to 10 AU/mL were considerate as positive results.

### Data sources, variables, and outcomes

All demographic and clinical data were prospectively collected in an ad hoc file. The sources of data and information were clinical records including all available information sources (i.e., medical and nursing assessments, administration records, operative checklists, laboratory examinations…), data from the hospital information system, and from the local death registry. Information on missing data are available on Table 3S (Supplementary material).

The outcomes we assessed were symptoms, mMRC dyspnea scale, neurological symptoms, overall mortality, COVID-related re-hospitalization, IgG antibody presence, 6 min walking test, and CT images’ scores.

### Statistical analyses

The frequency and percentage of occurrence were used to express dichotomous variables or scores. The Shapiro–Wilk test was used to ensure that continuous variables were normal, and they were then expressed as mean and standard deviation or median and first and third quartiles (Q1-Q3), as appropriate. The Fisher's exact test for dichotomous variables or scores, the unpaired t-test for continuous normally distributed variables, and the U-Mann Whitney test for continuous non-normally distributed variables were used to test differences between groups. We used a stratified analysis to address the study's secondary goal, dividing patients into two subgroups: no oxygen or low flow oxygen (6 l/min) and high flow oxygen (> 6 l/min) or HFNC or NIV or IMV patients. We used univariate logistic regression models to see if patients who had more respiratory needs during their hospital stay had a higher risk of having poorer outcomes at follow-up. As a result, for each outcome, we calculated the Odds Ratio (OR) for HFNC, NIV, or IMV patients compared to low-flow oxygen or patients who did not receive treatment. Finally, we performed multivariate logistic regression analyses. The adjustment was made for variables that reached a p-value < 0.10 in the baseline characteristics comparison. Regardless, the Brescia-COVID Respiratory Severity Scale (BCRSS) and the PiO2/FiO2 ratio were not adjusted because they were only used in the decision-making process for the respiratory treatment to be given to the patient. A p-value < 0.05 was considered to be statistically significant. Statistical analyses were performed using the Stata software (StataCorp, College Station, Texas USA).

## Results

### Baseline characteristics

**Figure 1:**
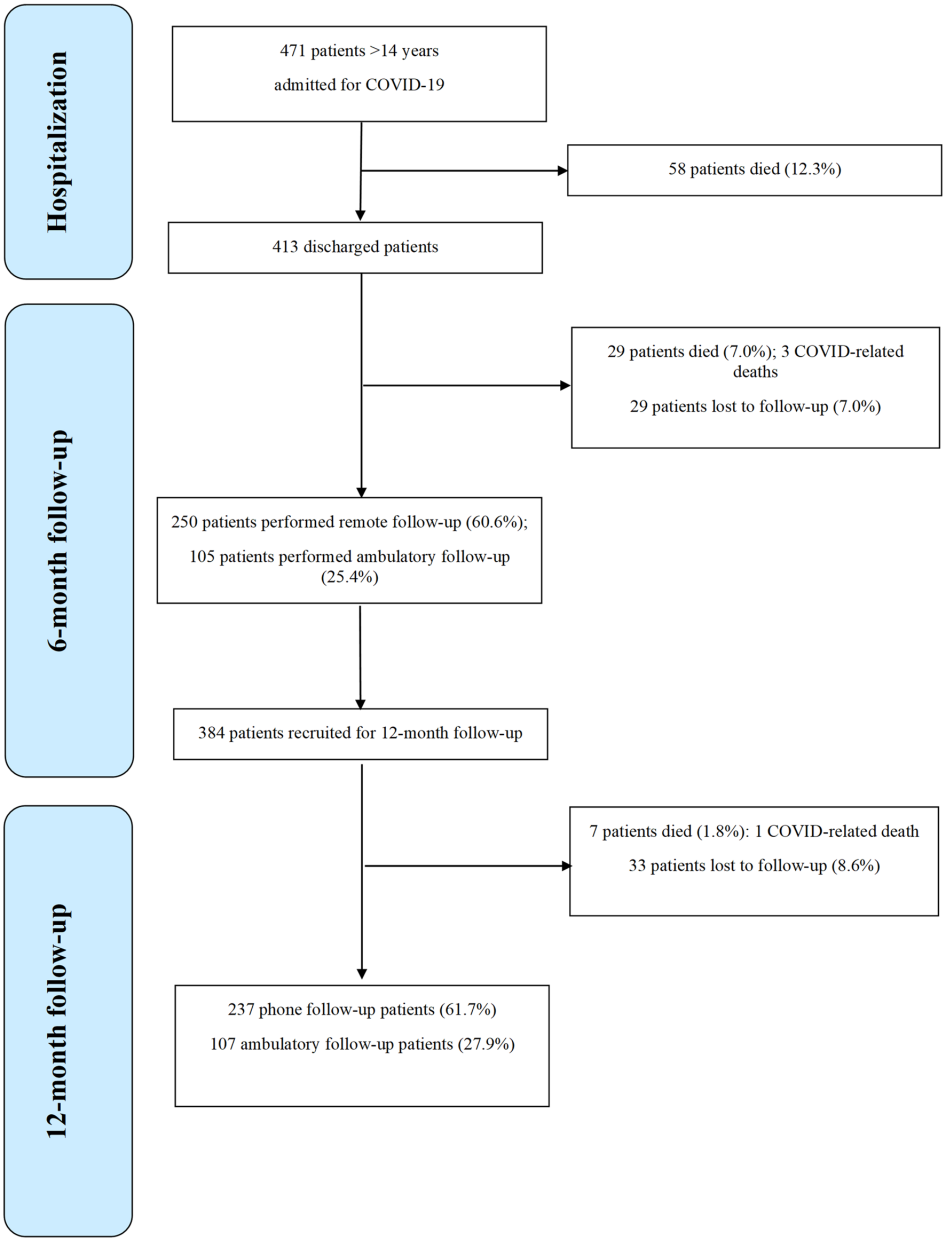
Patients’ flow diagram.

The demographic and clinical characteristics of the 471 eligible hospitalized patients who were admitted to hospital with COVID-19 are shown in Table 1. In total, 63.8% (301) were male, 36.2% (170) were female, and the median age was 71 (first and third quartiles 58–81) years. During hospitalization, 80.9% (381 patients) had received no respiratory support or supplemental low-flow oxygen via a nasal tube or mask; 8.9% (42 patients) had received HFNC or NIV; and 10.2% (48 patients) had required IMV. Among the patients hospitalized, 12.3% (58 patients) died during their hospital stay, therefore 413 patients were prospectively enrolled after discharge for the six-month follow-up. The patients’ flow diagrams are shown in Fig. 1.

**Table 1:** Pooled, and stratified characteristics of hospitalized patients.

Characteristics	Pooled	Stratified	
	Patients n (%)	No supplemental oxygen or low-flow oxygen n (%)	HFNC or NIV or IMV
	471	381	90
Male, n (%)	301 (63.8)	225 (59.1)	76 (84.4)
Female, n (%)	170 (36.2)	156 (40.9)	14 (15.6)
Age, median (Q1–Q3) years	71 (58–81)	73 (57–82)	67 (59–74)
Age male, median (Q1–Q3) years	68 (56–78)	68 (56–79)	67 (59–73)
Age female, median (Q1–Q3) years	76 (62–85)	77 (63–86)	65 (57–79)
BCRSS at admission 0, n (%)	226 (48.1)	224 (58.8)	2 (2.2)
BCRSS at admission 1, n (%)	112 (23.8)	109 (28.6)	3 (3.4)
BCRSS at admission 2, n (%)	47 (10.0)	43 (11.3)	4 (4.5)
BCRSS at admission 3, n (%)	45 (9.6)	5 (1.3)	40 (44.9)
BCRSS at admission 4, n (%)	38 (8.1)	0 (0.0)	38 (42.7)
BCRSS at admission 5, n (%)	2 (0.4)	0 (0.0)	2 (2.3)
P/F ratio at admission, median (Q1–Q3)	310 (191–368)	333 (265–382)	128 (93–175)
P/F ratio < 300, n (%)	218 (46.5)	134 (35.3)	84 (94.4)
NEWS2 at admission, median (Q1–Q3)	4 (2–7)	3 (2–6)	8 (6–9)
NEWS2 0–4, n (%)	258 (54.9)	244 (64.0)	14 (15.7)
NEWS2 5–6, n (%)	83 (17.7)	68 (17.9)	15 (16.9)
NEWS2 ≥ 7, n (%)	129 (27.4)	69 (18.1)	60 (67.4)
**Comorbidities**			
Cardiovascular (including hyperension), n (%)	289 (61.5)	237 (62.2)	52 (58.4)
Diabetes, n (%)	82 (17.4)	67 (17.6)	15 (16.9)
Gastrointestinal, n (%)	73 (15.5)	56 (14.7)	17 (19.1)
Autoimmune, n (%)	62 (13.2)	55 (14.4)	7 (7.9)
Obesity, n (%)	55 (11.7)	37 (9.7)	18 (20.2)
Pulmonary, n (%)	57 (12.1)	48 (12.6)	9 (10.1)
Renal, n (%)	50 (10.6)	41 (10.8)	9 (10.1)
Cancer, n (%)	56 (11.9)	49 (12.9)	7 (7.9)
**Outcomes**			
Intra-hospital mortality, n (%)	58 (12.3)	46 (12.1)	12 (13.3)
Length of stay, median (Q1–Q3)	10 (6–16)	9 (6–14)	21 (13–33)
**Complications**		17 (4.5)	
Venous thromboembolism (pulmonary embolism–deep vein thrombosis), n (%)	28 (6.0)	8 (2.1)	11 (12.4)
Acute coronary syndrome, n (%)	10 (2.1)	15 (3.9)	2 (2.2)
Sepsis, n (%)	24 (5.1)	0 (0.0)	9 (10.1)
Guillian-Barrè syndrome, n (%)	1 (0.2)	1 (0.3)	1 (1.1)
Pneumothorax, n (%)	4 (0.8)	1 (0.3)	3 (3.4)
Pneumomediastinum, n (%)	6 (1.3)	17 (4.5)	5 (5.6)

*BCRSS * Brescia COVID Respiratory Severity Scale, *HFNC*  high-flow nasal cannula, *IMV*  invasive mechanical ventilation, *NEWS2*  National Early Warning Score 2, *NIV*  non-invasive ventilation, *P/F ratio*  PiO2/FiO2 ratio, *Q1*  first quartile, *Q3* third quartile.

### Stratified analysis

Table 1 shows also the baseline characteristics of hospitalized patients stratified by the type of respiratory support they received during their stay. In terms of gender (male 59.1% versus 84.4% for no supplemental/low-flow oxygen and HFNC/NIV/IMV, respectively, p < 0.01), median age (73 years for no supplemental/low-flow oxygen versus 67 years for HFNC/NIV/IMV, p < 0.01), and BCRSS (HFNC/NIV/IMV patients had the worst scores, as expected, p < 0.01), the two groups were not homogeneous. In terms of intra-hospital outcomes, HFNC/NIV/IMV patients had a longer stay than non-oxygen patients: 21 (13–33) days versus 9 (6–14) days, p < 0.01. In addition, HFNC/NIV/IMV patients had a higher rate of intra-hospital complications: venous thromboembolism (12.4% versus 4.5%, p < 0.01), sepsis (10.1% versus 3.9%, p = 0.03), pneumothorax (3.4% versus 0.3%, p < 0.01), and pneumomediastinum (5.6% versus 0.3%, p < 0.01). There were no statistically significant differences in intra-hospital mortality or other intra-hospital complications between the two groups.

### Six-month follow-up results

Out of 413 discharged patients, 29 patients died and 29 were lost to follow-up. In total, 250 patients completed remote follow-up and 105 patients completed ambulatory follow-up (Fig. 1).

The median age of the 29 dead patients was 82 years (first quartile 76, third quartile 88). Post-COVID respiratory failure and/or cachexia were the causes of the three COVID-related deaths. Other causes of death included multi-morbidity (15), cancer (3), heart failure (5), and other factors.

**Figure 2:**
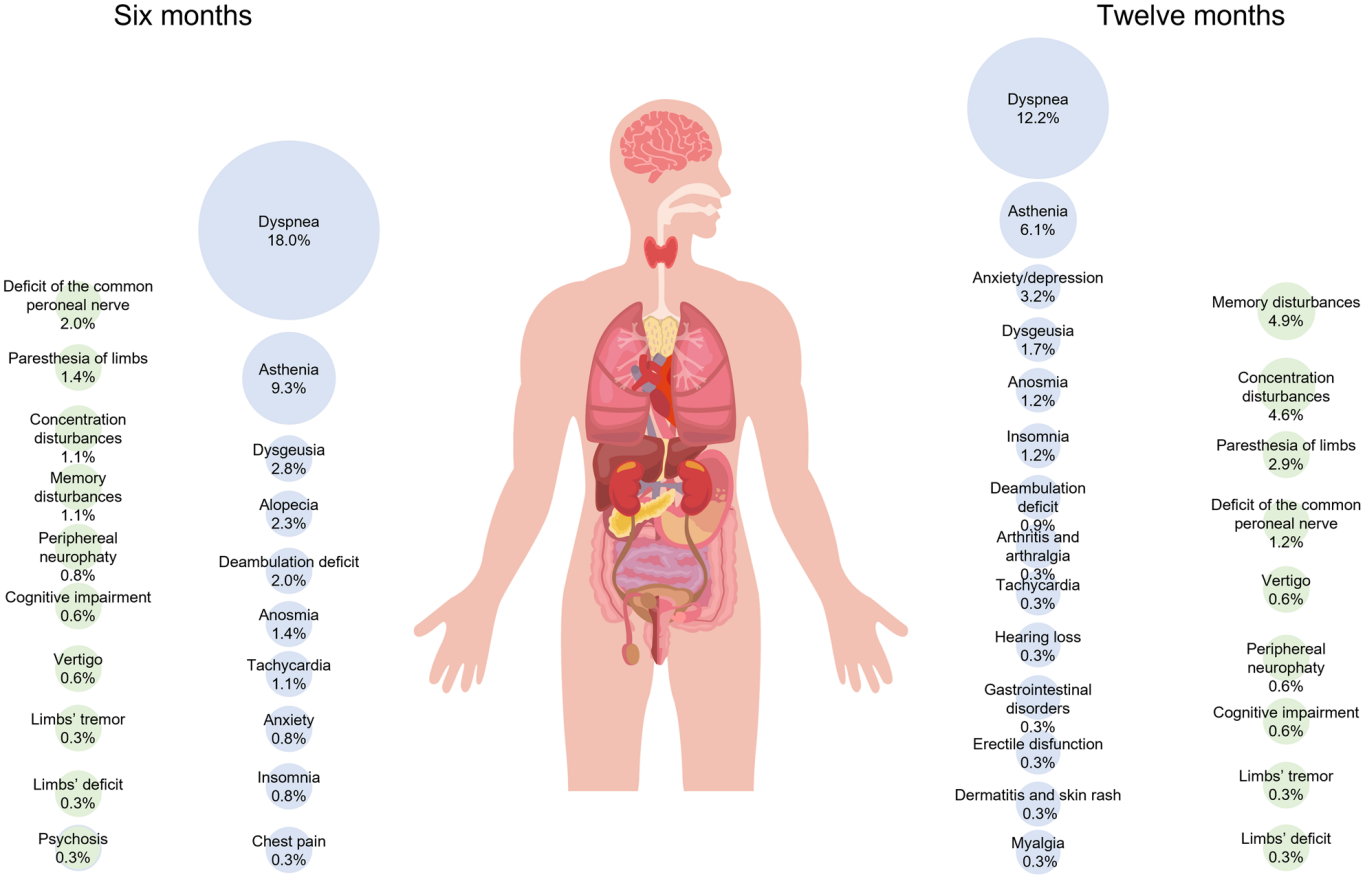
General (blue color) and neurological symptoms (green color) at 6 (left) and 12 months (right) after discharge for hospitalized SARS-CoV-2 patients.

Outcomes at 6-month were summarized in Table 2. Six months after discharge, 30.3% of patients suffered of any symptoms (displayed in Fig. 2, and analytically reported in Table 2S—Supplementary material). Furthermore, 18.0% of patients felt dyspnea and had altered or abnormal results on the mMRC scale (Table 2). A total of 6.2% of patients experienced neurological symptoms (see Fig. 2 and Table 2S for details). Globally, 22 different general symptoms/neurological symptoms were reported by patients. The COVID-related 6-month re-hospitalization was 2.6%, and overall mortality was 18.5%. The IgG were found in the 93.2% of the tested patients, while 12.4% of patients showed an altered or scarce walking test. At 6 months, 61.9% of the 105 patients studied had parenchymal abnormalities of the lung on chest imaging, with unilateral ground-glass opacity (GGO) in 9.5%, bilateral GGO in 49.5%, unilateral consolidation in 1.0%, and bilateral consolidation in 1.9% The interstitium abnormalities were recorded in 87.6% of patients, including unilateral thickening of the subpleural perilobular septa in 7.6% of patients, bilateral thickening was present in 30.5% of cases, 33.3% also with bilateral subpleural bronchiolectasis and 16.2% with bronchiectasis in addition to brochiolectasis.

**Table 2:** Pooled, and stratified by received respiratory support 6-month outcomes.

	Pooled	Stratified		OR (95% CI), p-value	aOR^#^ (95% CI), p-value
	N (%)	No supplemental oxygen or low-flow oxygen, n (%)	HFNC or NIV or IMV, n (%)		
	355	287	68		
**Outcomes**					
Symptoms	108/355 (30.3)	68 (23.8)	40 (57.1)	4.27 (2.48–7.38), < 0.01	4.00 (1.99–8.05), < 0.01
Dyspnea	64/355 (18.0)	39 (13.7)	25 (35.7)	3.50 (1.93–6.35), < 0.01	2.80 (1.28–6.16), 0.01
mMRC scale 0	292 (82.0)	247 (86.4)	45 (64.3)	*3.52 (1.94–6.37), < 0.01	*2.85 (1.30–6.25), < 0.01
mMRC scale 1	52 (14.6)	33 (11.5)	19 (27.1)		
mMRC scale 2	8 (2.3)	5 (1.8)	3 (4.3)		
mMRC scale 3	3 (0.8)	1 (1.3)	2 (2.9)		
mMRC scale 4	1 (0.3)	0 (0.0)	1 (1.4)		
Neurological symptoms	22/355 6.2)	6 (2.1)	16 (22.9)	13.78 (5.16–36.80), < 0.01	9.72 (2.78–34.00), < 0.01
COVID-related re-hospitalization	10/384 (2.6)	8/308 (2.6)	2/76 (2.6)	1.01 (0.21–4.87), 0.99	2.67 (0.34–20.87), 0.35
Overall 6-month mortality	87/471 (18.5)	73 (19.2)	14 (15.6)	0.78 (0.42–1.45), 0.43	0.53 (0.23–1.19), 0.12
IgG	177/190 (93.2)	121/131 (92.4)	56/59 (94.9)	1.54 (0.41–5.82), 0.52	1.27 (0.21–7.57), 0.79
Walking test 0	92/104 (87.6)	45 (91.8)	47 (84.0)	*2.15 (0.62–7.49), 0.23	*2.68 (0.48–15.03), 0.26
Walking test 1	12/104 (11.4)	4 (8.2)	8 (14.2)		
Walking test 2	1/104 (1.0)	0 (0.0)	1 (1.8)		
Parenchymal CT 0, 1, 2 (normal or mild damage)	102/105 (98.1)	42 (97.7)	60 (96.8)	1.43 (0.13–16.32), 0.77	4.29 (0.15–123.07), 0.40
Parenchymal CT 3, 4 (moderate to severe damage)	3/105 (2.9)	1 (2.3)	2 (3.2)		
Interstitial CT 0, 1, 2 (normal or mild damage)	53/105 (50.5)	31 (70.5)	22 (37.1)	4.04 (1.77–9.25), < 0.01	2.73 (0.90–8.28), 0.08
Interstitial CT 3, 4 (moderate to severe damage)	52/105 (49.5)	13 (29.5)	39 (62.9)		

*CT*  computed tomography, *HFNC*  high-flow nasal cannula, *IgG*  immunoglobulin G, *IMV* invasive mechanical ventilation, *mMRC*  Modified Medical Research Council, *NIV*  non-invasive mechanical ventilation, *TC*  computed tomography.

*For every 1 point increase.

^#^Adjusted for gender, age, obesity, and NEWS2 (National Early Warning Score 2) at admission.

### Stratified analysis

In terms of outcomes stratified by respiratory support received during hospitalization (Table 2), patients treated with HFNC, NIV, or IMV had a four-fold higher risk of experiencing symptoms at 6 months than those treated with no supplemental or low-flow oxygen: crude OR = 4.27 (95%CI: 2.48–7.38), p < 0.01, adjusted OR = 4.00 (95%CI: 1.99–8.05), p < 0.01; had almost three-fold higher risk of having dyspnea: crude OR = 3.50 (95%CI: 1.93–6.35), p < 0.01, adjusted OR = 2.80 (95%CI: 1.28–6.16), p < 0.01; had nearly ten-fold higher risk of neurological symptoms: crude OR = 13.78 (95%CI: 5.16–36.80), p < 0.01, adjusted OR = 9.72 (95%CI: 2.78–34.00), p < 0.01; and had higher risk of moderate to severe damage in the interstitial CT images: crude OR = 4.04 (95%CI: 1.77–9.25), p < 0.01, adjusted OR = 2.73 (95%CI:0.90–8.28), p = 0.08. For 6-month COVID-related re-hospitalization, overall 6-month mortality, presence of IgG antibodies, walking test, and parenchymal damages in chest images, no statistically significant differences were found between the two groups.

### Twelve-month follow-up results

By the 12-month follow-up, 7 more patients died, 237 patients performed a phone follow-up, 107 patients performed an ambulatory follow-up, and 33 patients were lost to follow-up (Fig. 1). Outcomes at 12 months were summarized in Table 3.

**Table 3:** Pooled, and stratified by received respiratory support 12-month outcomes.

	Pooled	Stratified		OR (95% CI), p-value	aOR^#^ (95% CI), p-value
	N (%)	No supplemental oxygen or low-flow oxygen, n (%)	HFNC or NIV or IMV, n (%)		
	344	276	68		
**Outcomes**					
Symptoms	87/344 (25.3)	57 (20.6)	30 (44.1)	3.03 (1.73–5.31), < 0.01	3.66 (1.73–7.74), < 0.01
Dyspnea	42/345 (12.2)	29 (10.5)	13 (18.8)	1.98 (0.97–4.04), 0.06	1.87 (0.73–4.76), 0.19
mMRC scale 0	303 (87.8)	247 (89.5)	56 (81.1)	*1.98 (0.97–4.04), 0.06	*1.87 (0.73–4.76), 0.19
mMRC scale 1	32 (9.3)	23 (8.3)	9 (13.0)		
mMRC scale 2	6 (1.7)	4 (1.5)	2 (2.9)		
mMRC scale 3	3 (0.9)	2 (0.7)	1 (1.5)		
mMRC scale 4	1 (0.3)	0 (0.0)	1 (1.5)		
Neurological symptoms	35/345 (10.1)	15 (5.4)	20 (29.0)	7.10 (3.40–14.82), < 0.01	8.96 (3.22–24.90), < 0.01
COVID-related re-hospitalization	5/377 (1.3)	5/302 (1.7)	0/75 (0.0), (p^§^ = 0.59)	Not performable	Not performable
Overall 12-month mortality	94/471 (20.0)	79/381 (20.7)	15/90 (16.7)	0.76 (0.42–1.40), 0.39	0.54 (0.24–1.20), 0.13
IgG	192/224 (85.7)	137/161 (85.6)	55/62 (85.9)	1.03 (0.45–2.36), 0.95	1.82 (0.56–5.87), 0.32
Vaccinated	132/322 (41.0)				
Walking test 0	7/11 (63.6)	6 (75.0)	1 (33.3)	*6.00 (0.33–107.42), 0.22	Not performable
Walking test 1	4/11 (36.4)	2 (25.0)	2 (66.7)		
Walking test 2	0/11 (0.0)	0 (0.0)	0 (0.0)		
Parenchymal CT 0, 1, 2 (normal or mild damage)	46/47 (97.9)	12 (100.0)	34 (97.1)	Not performable	Not performable
Parenchymal CT 3, 4 (moderate to severe damage)	1/47 (2.1)	0 (0.0)	1 (2.9), (p^§^ = 1.00)		
Interstitial CT 0, 1, 2 (normal or mild damage)	10/47 (21.3)	4 (33.3)	6 (17.1)	2.42 (0.55–10.70), 0.25	0.83 (0.07–9.15), 0.88
Interstitial CT 3, 4 (moderate to severe damage)	37/47 (78.7)	8 (66.7)	29 (82.9), 0.25		

*CT*  computed tomography, *HFNC*  high-flow nasal cannula, *IgG*  immunoglobulin G, *IMV*  invasive mechanical ventilation, *mMRC*  Modified Medical Research Council, *NIV*  non-invasive mechanical ventilation, *TC*  computed tomography.

*For every 1 point increase.

^#^Adjusted for gender, age, obesity, and NEWS2 (National Early Warning Score 2) at admission.

^§^Fischer exact test.

The median age of the 7 dead patients was 80 years (first quartile 69, third quartile 95). Post-covid respiratory failure caused one COVID-related death. Other causes of death included multi-morbidity (3), cancer (2), and heart failure (1).

Twelve months after discharge, 25.3% of patients suffered of any symptoms (see Fig. 2, and Table 2S for details); 12.2% of patients felt dyspnea, and had altered or abnormal results on the mMRC scale (Table 3). In total, the 10.1% of patients experienced neurological symptoms (see Fig. 2, and Table 2S for details). Globally, 28 different symptoms/neurological symptoms were reported by patients. The COVID-related 12-month re-hospitalization was 1.3%, and overall mortality was 20.0%. IgG antibodies were found in the 85.7% of the patients tested and 41.0% of the patients followed up had been vaccinated. Among the patients investigated with CT images at 12 months (47), GGO was still present in 48.9% of patients (with bilateral damage in 46.8% of cases), while 2.1% continued to show consolidation (score > 2). Interstitium abnormalities in this group of patients included thickening of the subpleural perilobular septa in 95.7% of patients, bilateral thickening was found in 12.8% of cases, 51.1% also with bilateral subpleural bronchiolectasis and 27.7% with bronchiectasis in addition to brochiolectasis. Among patients followed up with CT at 6 months, it could be estimated that any form of GGO was found in 22.6% of cases, while interstitial severe damages (score > 2) were still present in 34.9% of patients followed up with CT at 6 months.

### Stratified analysis

Table 3 also shows the outcomes by type of respiratory support received during hospitalization. At 12 months, patients who received HFNC, NIV, or IMV had a three-fold higher risk of experiencing symptoms than those who received no supplemental oxygen or low-flow oxygen: crude OR = 3.03 (95%CI: 1.73–5.31), p < 0.01, adjusted OR = 3.66 (95%CI: 1.73–7.74), p < 0.01; had almost nine-fold higher risk of suffering of neurological symptoms: crude OR = 7.10 (95%CI:3.40–14.82), p < 0.01, adjusted OR = 8.96 (95%CI: 3.22–24.90), p < 0.01. Dyspnea, 12-month COVID-related re-hospitalization, overall 12-month mortality, presence of IgG antibodies, walking test, and parenchymal and interstitial damages in chest images showed no statistically significant differences between the two groups.

### Ethics statement

The study protocol was approved by the ethic committee of the Autonomous Province of Trento (reference number 4659) and followed the Declaration of Helsinki Ethical Principles for Medical Research Involving Human Subjects. Informed consent was obtained from all individual participants for whom identifying information is included in this article.

## Discussion

We implemented a long-term stratified follow-up path that adhered to the international proposed clinical guidance for the assessment and management of COVID-19 patients^7,8,36,37^, in the context of the limited availability of resources and the operational pressure faced by hospitals during the pandemic. This project was part of a broad set of strategies and paths implemented within the Healthcare Trust of the Autonomous Province of Trento for reorganizing care and managing patients at a distance, including routine services and the continuation of care after hospital discharge^38^, as well assessing the impact of pandemic burden and related change in practice^39^. In our study, we targeted the same cohort of patients (excluding six patients with multiple admissions) to investigate in-hospital outcomes during the early phase of the COVID-19 pandemic^40^.

Overall, in our study any long COVID symptoms were reported at 6 months in 30.3% of patients and at 12 months in 25.3%, but we noted an increased number of different symptoms at 12 months. Alterations in the mMRC scale were reported in 18.0% of patients at 6 months and in 12.2% of patients at 12 months. The frequency of neurocognitive symptoms increased from 6.2% to 10.1% from the 6-month to the 12-month timepoints. At both 6 and 12 months, general and neurocognitive symptoms were markedly more common in patients who had been treated with non-invasive or invasive mechanical ventilation during hospitalization. Furthermore, patients who received tracheal intubation, HFNC, or NIV had a significantly higher risk of developing dyspnea at 6 months than those who received no therapy or low-flow oxygen.

Published studies investigating the highly heterogeneous and poorly understood post-COVID-19 syndrome show the relevance of medical and psychological sequelae for several months after active infection, with more than 50 long-term effects of COVID-19 having been reported^41^. Pooled prevalence data show that the 10 most prevalent symptoms are fatigue, shortness of breath, muscle pain, joint pain, headache, cough, chest pain, altered smell, altered taste, and diarrhea^42^. The increasingly evident long-term neurological effects include the impact of the virus on cognition, autonomic function, and mental wellbeing^43^. Patients with long COVID present with prolonged multisystem involvement and significant disability^13^. The development of long COVID symptoms may be linked to symptomatic COVID-19 infection, hospitalization (with mechanical ventilation being required), severity of illness, and sex (women may have a higher incidence)^44–46^. However, any patient with COVID-19 may develop long COVID, regardless of the severity of their infection and the intensity of the treatment they received^45^.

The few published prospective studies using a 12-month timepoint providing an overview of the clinical symptoms and quality of life of adult patients show that, although decreasing over time, a meaningful portion of patients still report persistent symptoms one year after infection^20,22–25^. Overall, our findings are in line with such results, although with a lower proportion of patients experiencing persistent symptoms, with a meaningful proportion of patients of experiencing dyspnea and patterns of neurologic symptoms. Clinical impairment can persist at least until one year after COVID-19 symptom onset and reduce patients’ quality of life significantly^22^. The persistence of neuropsychiatric long COVID symptoms (which can reduce quality of life significantly) 1 year after COVID-19 symptom onset may be partially explained by the influence of the extended pandemic situation and consequent psychological impact^22^.

Our findings seem consistent with the literature showing the persistence of chest imaging manifestations months after hospitalization for COVID-19 pneumonia^12,47–49^ and the persistence (although decreasing over time) of pulmonary alterations up to 12 months later^21,25^. At 6 months, we found an association between lung structural abnormalities and disease severity, but not at 12 months. Lung imaging patterns at 12 months may be associated with lung diffusion impairment, although further studies are needed to explore the effect of these persistent abnormalities on physical function and quality of life^25^. In our study, we found a decreased detection of IgG antibodies in SARS-CoV-2-infected patients, dropping from 93.2% at six months to 85.7% at 12 months. The detectability of antibodies 1 year after infection, although with different temporal trends and magnitudes, seems to confirm the findings of other studies that have carried out comparable long-term follow-ups^50–52^. The relationship between the antibody level and protection against COVID-19 is still unclear; however, the one-year follow-up data show that patients who have recovered from COVID-19 have a very low risk of reinfection. Natural immunity to SARS-CoV-2 appears to confer a protective effect for at least a year^53^. The rate of full vaccine coverage at 12 months seem to mirror the progress of the Italian vaccination campaign, which is still underway at the timeframe of the writing, reflecting the need of specific logistic organization^54–56^.

All in all, our study shows the high clinical burden of long COVID-19 12 months after acute infection and affirms the importance of understanding the natural course of long COVID as a long-term chronic condition with symptoms persisting beyond 12 months after the onset of illness. Identifying patients at major risk of sequelae from the early post-acute phase; setting up appropriate and patient-centered pathways supported by online support tools; and the implementation of surveillance systems and specialized multidisciplinary care, including rehabilitation, are critical in order to understand and treat patients suffering from long COVID^42,57–60^.

The development and implementation of clinical guidelines^61^ as well as use of tools and methods for Health Technology Assessment^62,63^ in order to evaluate the effects of decisions and actions related to resource allocation, models of care, professional practice, drugs, and medical devices in response to the complex and evolving challenges of long COVID may enable value-based decisions to be made^64^.

## Strengths and limitations

To the best of our knowledge, our research represents one of the few 12-month follow-up studies on a cohort of COVID-19 patients infected at the very beginning of the pandemic, addressing a unique mix of clinical findings and including the investigation of a broad range of clinical symptoms, serum antibodies titers, pulmonary functions, and CT imaging and vaccination statuses.

The strength of our study is its long-term follow-up of a well-characterized patient population from the first peak of the pandemic in Italy (with almost no missing data, see Table 3S, and no risk of any selection bias) within a real world standardized pathway in which we prioritized instrumental assessments and investigations directed at survivors by exploiting a telehealth solution to reduce ambulatory burden and patient risk. Following the appropriateness principle, computed tomography was performed at 12 months only on survivors who had interstitial abnormalities of degree 3 and 4 at 6 months, and the Six-Minute Walking Test was performed at 12 months only on patients who had altered test results at 6 months. Considering the diversity of the initial target population and the duration of the pathway, we believe that our drop-out rate was very low, which may be attributable to the benefit of engaging patients within a structured and patient-oriented follow-up path.

We acknowledge the potential bias caused by not performing the same investigations on all patients prospectively followed by either comparing cohort one and two as well as within cohort two (see above). Furthermore, our findings apply to a population in which we did not conduct a clinical assessment to rule out the presence of any pre-existing clinical conditions prior to COVID-19 infection or hospitalization. Since early COVID, both the characteristics of the presenting patient and the clinical management have changed. The potential for patient population selection bias, the lack of a comparison (control) group, the lack of standardized validated tools for reporting persistent symptoms after hospitalization due to COVID-19, and the study's single-center, non-blinded, and non-randomized design may limit the generalizability of our findings.

## Conclusions

Our study may provide valuable information enabling relevant professionals to understand the clinical needs of long COVID patients and identify practical and feasible approaches for the routine follow-up and management of patients with long-term COVID-19 complications. Large and long-ranging observational studies and clinical trials for investigating long-term sequelae, as well as follow-up pathways for looking after people with long-term complications after acute COVID-19, should be considered.

## Supplementary Information

Supplementary Tables.

## Data Availability

Data are available on reasonable requests.

## References

[1] World Health Organization. *Coronavirus Disease (COVID-19)—World Health Organization*. https://www.who.int/emergencies/diseases/novel-coronavirus-2019.

[2] World Health Organization. *Critical Preparedness, Readiness and Response Actions for COVID-19: Interim Guidance, 22 March 2020*. https://apps.who.int/iris/handle/10665/331511 (2020).

[3] Wu Z, McGoogan JM (2020). Characteristics of and important lessons from the coronavirus disease 2019 (COVID-19) outbreak in China: Summary of a report of 72 314 cases from the Chinese Center for Disease Control and Prevention. JAMA.

[4] Scherlinger M (2021). Refining “long-COVID” by a prospective multimodal evaluation of patients with long-term symptoms attributed to SARS-CoV-2 infection. Infect. Dis. Ther..

[5] Nalbandian A (2021). Post-acute COVID-19 syndrome. Nat. Med..

[6] Datta SD, Talwar A, Lee JT (2020). A proposed framework and timeline of the spectrum of disease due to SARS-CoV-2 infection: Illness beyond acute infection and public health implications. JAMA.

[7] *COVID-19 Rapid Guideline: Managing the Long-Term Effects of COVID-19*. (National Institute for Health and Care Excellence (UK), 2020). 33555768

[8] CDC. Healthcare Workers. *Centers for Disease Control and Prevention.* https://www.cdc.gov/coronavirus/2019-ncov/hcp/clinical-care/post-covid-conditions.html (2020).

[9] Venkatesan P (2021). NICE guideline on long COVID. Lancet Respir. Med..

[10] The Lancet, null. Facing up to long COVID. *Lancet Lond. Engl.* **396**, 1861 (2020). 10.1016/S0140-6736(20)32662-3 PMC7834723 33308453

[11] Logue JK (2021). Sequelae in adults at 6 months after COVID-19 infection. JAMA Netw. Open.

[12] Huang C (2021). 6-month consequences of COVID-19 in patients discharged from hospital: A cohort study. Lancet Lond. Engl..

[13] Davis HE (2021). Characterizing long COVID in an international cohort: 7 months of symptoms and their impact. EClinicalMedicine.

[14] Havervall S (2021). Symptoms and functional impairment assessed 8 months after mild COVID-19 among health care workers. JAMA.

[15] Carfì A, Bernabei R, Landi F, Gemelli Against COVID-19 Post-Acute Care Study Group (2020). Persistent symptoms in patients after acute COVID-19. JAMA.

[16] Munblit D (2021). Incidence and risk factors for persistent symptoms in adults previously hospitalized for COVID-19. Clin. Exp. Allergy J. Br. Soc. Allergy Clin. Immunol..

[17] Akbarialiabad H (2021). Long COVID, a comprehensive systematic scoping review. Infection.

[18] Günster C (2021). 6-month mortality and readmissions of hospitalized COVID-19 patients: A nationwide cohort study of 8,679 patients in Germany. PLoS ONE.

[19] Boscolo-Rizzo P (2021). Sequelae in adults at 12 months after mild-to-moderate coronavirus disease 2019 (COVID-19). Int. Forum Allergy Rhinol..

[20] Wu X (2021). 3-month, 6-month, 9-month, and 12-month respiratory outcomes in patients following COVID-19-related hospitalisation: a prospective study. Lancet Respir. Med..

[21] Yan X (2021). Follow-up study of pulmonary function among COVID-19 survivors 1 year after recovery. J. Infect..

[22] Seeßle J (2021). Persistent symptoms in adult patients one year after COVID-19: a prospective cohort study. Clin. Infect. Dis..

[23] Budhiraja S (2021). Long term health consequences of COVID-19 in hospitalized patients from North India: A follow up study of up to 12 months. Medrxiv..

[24] Sigfrid L (2021). Long Covid in adults discharged from UK hospitals after Covid-19: A prospective, multicentre cohort study using the ISARIC WHO clinical characterisation protocol. Lancet Reg. Health Eur..

[25] Huang L (2021). 1-year outcomes in hospital survivors with COVID-19: A longitudinal cohort study. Lancet Lond. Engl..

[26] World Health Organization (2020). Clinical management of severe acute respiratory infection (SARI) when COVID-19 disease is suspected. Interim guidance. Pediatr. Med. Rodz..

[27] Italian Health Ministry. General Directorate of Health Prevention. *Circular letter no. 11715 of April 1st 2021. Pandemia di COVID-19—Aggiornamento delle indicazioni sui test diagnostici e sui criteri da adottare nella determinazione delle priorità. Aggiornamento delle indicazioni relative alla diagnosi di laboratorio*.

[28] Duca A, Piva S, Focà E, Latronico N, Rizzi M (2020). Calculated decisions: Brescia-COVID respiratory severity scale (BCRSS)/algorithm. Emerg. Med. Pract..

[29] Italian Health Ministry. General Directorate of Health Prevention. *Circular letter no. 8248 of March 3rd 2021. Vaccinazione dei soggetti che hanno avuto un’infezione da SARS-CoV-2*.

[30] Genecand L (2021). Diagnostic and therapeutic management of medium and long-term sequelae of SARS-CoV-2 infection. Rev. Med. Suisse.

[31] Bestall JC (1999). Usefulness of the Medical Research Council (MRC) dyspnoea scale as a measure of disability in patients with chronic obstructive pulmonary disease. Thorax.

[32] Enright PL, Sherrill DL (1998). Reference equations for the six-minute walk in healthy adults. Am. J. Respir. Crit. Care Med..

[33] Wu J (2020). Chest CT findings in patients with coronavirus disease 2019 and its relationship with clinical features. Invest. Radiol..

[34] Kwee TC, Kwee RM (2020). Chest CT in COVID-19: What the radiologist needs to know. Radiogr. Rev. Publ. Radiol. Soc. N. Am. Inc.

[35] Infantino M (2020). Diagnostic accuracy of an automated chemiluminescent immunoassay for anti-SARS-CoV-2 IgM and IgG antibodies: An Italian experience. J. Med. Virol..

[36] Raghu G, Wilson KC (2020). COVID-19 interstitial pneumonia: Monitoring the clinical course in survivors. Lancet Respir. Med..

[37] Greenhalgh T, Knight M, A’Court C, Buxton M, Husain L (2020). Management of post-acute covid-19 in primary care. BMJ.

[38] Testa S (2021). Implementation of tele visit healthcare services triggered by the COVID-19 emergency: The Trentino Province experience. Z. Gesundheitswissenschaften J. Public Health.

[39] Ciarleglio FA (2021). The negative effects of COVID-19 and national lockdown on emergency surgery morbidity due to delayed access. World J. Emerg. Surg. WJES.

[40] Rigoni M, Torri E, Nollo G, Delle Donne L, Cozzio S (2021). NEWS2 is a valuable tool for appropriate clinical management of COVID-19 patients. Eur. J. Intern. Med..

[41] Lopez-Leon S (2021). More than 50 long-term effects of COVID-19: a systematic review and meta-analysis. Sci. Rep..

[42] Aiyegbusi OL (2021). Symptoms, complications and management of long COVID: a review. J. R. Soc. Med..

[43] Jesuthasan A, Massey F, Manji H, Zandi MS, Wiethoff S (2021). Emerging potential mechanisms and predispositions to the neurological manifestations of COVID-19. J. Neurol. Sci..

[44] Baratta JM, Tompary A, Siano S, Floris-Moore M, Weber DJ (2021). Postacute sequelae of COVID-19 infection and development of a physiatry-led recovery clinic. Am. J. Phys. Med. Rehabil..

[45] Crook H, Raza S, Nowell J, Young M, Edison P (2021). Long covid-mechanisms, risk factors, and management. BMJ.

[46] Fernández-de-Las-Peñas C (2021). Prevalence of post-COVID-19 symptoms in hospitalized and non-hospitalized COVID-19 survivors: A systematic review and meta-analysis. Eur. J. Intern. Med..

[47] Blanco J-R (2021). Pulmonary long-term consequences of COVID-19 infections after hospital discharge. Clin. Microbiol. Infect. Off. Publ. Eur. Soc. Clin. Microbiol. Infect. Dis..

[48] Sideris GA (2021). Imaging in the COVID-19 era: Lessons learned during a pandemic. World J. Radiol..

[49] Zhang S (2021). Eight months follow-up study on pulmonary function, lung radiographic, and related physiological characteristics in COVID-19 survivors. Sci. Rep..

[50] Laing ED (2021). SARS-CoV-2 antibodies remain detectable 12 months after infection and antibody magnitude is associated with age and COVID-19 severity. medrxiv.

[51] Xiao K (2021). Antibodies can last for more than 1 year after SARS-CoV-2 infection: A follow-up study from survivors of COVID-19. Front. Med..

[52] Haveri A (2021). Persistence of neutralizing antibodies a year after SARS-CoV-2 infection. medrxiv..

[53] Vitale J (2021). Assessment of SARS-CoV-2 reinfection 1 year after primary infection in a population in Lombardy, Italy. JAMA Intern. Med..

[54] COVID-19 Vaccine Tracker | European Centre for Disease Prevention and Control. https://vaccinetracker.ecdc.europa.eu/public/extensions/COVID-19/vaccine-tracker.html#uptake-tab.

[55] Pilati F, Tronconi R, Nollo G, Heragu SS, Zerzer F (2021). Digital twin of COVID-19 mass vaccination centers. Sustainability.

[56] Nollo, G., Pilati, F, F., Tronconi, R. & Rigoni, M. Industry 4.0 at the service of public health against the COVID-19 pandemic. *Disaster Med. Public Health Prep.* **In press**,. 10.1017/dmp.2021.286 PMC8529351 34498556

[57] Garg M (2021). The conundrum of ‘long-COVID-19’: A narrative review. Int. J. Gen. Med..

[58] Menges D (2021). Burden of post-COVID-19 syndrome and implications for healthcare service planning: A population-based cohort study. PLoS ONE.

[59] Rajan, S. *et al. In the Wake of the Pandemic: Preparing for Long COVID*. (European Observatory on Health Systems and Policies, 2021). 33877759

[60] Herrera JE (2021). Multidisciplinary collaborative consensus guidance statement on the assessment and treatment of fatigue in postacute sequelae of SARS-CoV-2 infection (PASC) patients. PM R.

[61] Schünemann HJ (2020). Using GRADE in situations of emergencies and urgencies: certainty in evidence and recommendations matters during the COVID-19 pandemic, now more than ever and no matter what. J. Clin. Epidemiol..

[62] Miglietta A (2021). Health technology assessment applied to emergency preparedness: a new perspective. Int. J. Technol. Assess. Health Care.

[63] Grossi A (2021). Hospital contextual factors affecting the implementation of health technologies: A systematic review. BMC Health Serv. Res..

[64] Torri E, Nollo G (2020). Public health decision-making in the real world: Four points to reshape it after COVID-19. Disaster Med. Public Health Prep..

