# Supplementary material for: “Long COVID” results after hospitalization for SARS-CoV-2 infection
Source: Sci Rep. 2022 Jun 10;12:9581. doi: 10.1038/s41598-022-13077-5 (PMC9185134; doi:10.1038/s41598-022-13077-5)
Supplement: Supplementary file 1 — Supplementary Tables. [file 41598_2022_13077_MOESM1_ESM.docx]

Table 1S Supplementary material. COVID-19 follow-up questionnaire.

| **Questions** | **After 6 months**  **Yes/no** | **After 12 months**  **Yes/no** |
| --- | --- | --- |
| Have you had any symptoms since discharge? |  |  |
| Asthenia |  |  |
| Dyspnea (mMRC scale in addition) |  |  |
| Cough |  |  |
| Chest pain or tachycardia |  |  |
| Insomnia - Depression - Anxiety |  |  |
| Difficulty in memory and/or concentration |  |  |
| Taste and/or olfactory alterations |  |  |
| Any other symptoms? |  |  |
| Have you had any neurological alterations? |  |  |
| Have you been vaccinated for SARS-CoV-2?* |  |  |

Legend: * Only for 12-months follow-up; mMRC=modified British Medical Research Council

Table 2S Supplementary material. General and neurological symptoms for SARS-CoV-2 patients 6 and 12 months after discharge.

| **Six months** | | **Twelve months** | |
| --- | --- | --- | --- |
| **General symptoms** | n (%) | **General symptoms** | n (%) |
| Dyspnea | 64/355 | Dyspnea | 42/345 |
| Asthenia | 33/355 | Asthenia | 21/345 |
| Alopecia | 8/355 | Alopecia | 0/345 |
| Dysgeusia | 10/355 | Dysgeusia | 6/345 |
| Anosmia | 5/355 | Anosmia | 4/345 |
| Tachycardia | 4/355 | Tachycardia | 1/345 |
| Anxiety | 3/355 | Anxiety/Depression | 11/345 |
| Insomnia | 3/355 | Insomnia | 4/345 |
| Deambulation deficit | 7/355 | Deambulation deficit | 3/345 |
| Chest pain | 1/355 | Chest pain | 0/345 |
|  |  | Hearing loss | 1/345 |
|  |  | Gastrointestinal disorders | 1/345 |
|  |  | Erectile disfunction | 1/345 |
|  |  | Dermatitis and skin rash | 1/345 |
|  |  | Myalgia | 1/345 |
|  |  | Arthritis and arthralgia | 2/345 |
|  |  |  |  |
| **Neurological symptoms** |  | **Neurological symptoms** |  |
| Deficit of the common peroneal nerve | 7/355 | Deficit of the common peroneal nerve | 4/345 |
| Paresthesia of limbs | 5/355 | Paresthesia of limbs | 10/345 |
| Cognitive impairment | 2/355 | Cognitive impairment | 2/345 |
| Vertigo | 2/355 | Vertigo | 2/345 |
| Concentration disturbances | 4/355 | Concentration disturbances | 16/345 |
| Memory disturbances | 4/355 | Memory disturbances | 17/345 |
| Periphereal sensorial neurophaty | 1/355 | Periphereal sensorial neurophaty | 1/345 |
| Axonal polyneuropathy | 1/355 | Axonal polyneuropathy | 1/345 |
| Neuropathy | 1/355 | Neuropathy | 0/345 |
| Limbs’ tremor | 1/355 | Limbs’ tremor | 1/345 |
| Limbs’ deficit | 1/355 | Limbs’ deficit | 1/345 |
| Psychosis | 1/355 | Psychosis | 0/345 |

Table 3S Supplementary material. Focus on missing data.

| **Characteristics** | **Missing data n (%)** |
| --- | --- |
|  | 471 |
| Male/Female, n (%) | 0 (0.0) |
| Age, n (%) | 1 (0.2) |
| Age male, n (%) | 1 (0.2) |
| Age female, n (%) | 0 (0.0) |
| BCRSS at admission, n (%) | 1 (0.2) |
| P/F ratio at admission, n (%) | 2 (0.4) |
| NEWS2 at admission, n (%) | 1 (0.2) |
| **Comorbidities:** |  |
| Cardiovascular (including hyperension), n (%) | 1 (0.2) |
| Diabetes, n (%) | 1 (0.2) |
| Gastrointestinal, n (%) | 1 (0.2) |
| Autoimmune, n (%) | 1 (0.2) |
| Obesity, n (%) | 1 (0.2) |
| Pulmonary, n (%) | 1 (0.2) |
| Renal, n (%) | 1 (0.2) |
| Cancer, n (%) | 1 (0.2) |
| **Received respiratory support:** |  |
| No supplemental oxygen or low-flow oxygen, n (%) | 0 (0.0) |
| HFNC or NIV, n (%) | 0 (0.0) |
| IMV, n (%) | 0 (0.0) |
| **Outcomes:** |  |
| Intra-hospital mortality, n (%) | 0 (0.0) |
| Length of stay, n (%) | 17 (3.6) |
| **Complications:** |  |
| Venous thromboembolism (pulmonary embolism – Deep vein thrombosis), n (%) | 3 (0.6) |
| Acute coronary syndrome, n (%) | 3 (0.6) |
| Sepsis, n (%) | 3 (0.6) |
| Guillian-Barrè syndrome, n (%) | 3 (0.6) |
| Pneumothorax, n (%) | 3 (0.6) |
| Pneumomediastinum, n (%) | 3 (0.6) |
| 6-month outcomes, n (%) excluding 29 patients lost to follow-up | 1 (0.2) |
| 12-month outcomes, n (%)excluding 33 patients lost to follow-up | 1 (0.2) |
| 6-month lost to follow-up, n (%) | 29 (7.0) |
| 12-month lost to follow-up, n (%) | 33 (8.6) |

Legend: BCRSS= Brescia COVID Respiratory Severity Scale; HFNC= High-Flow Nasal Cannula; IMV= Invasive Mechanical Ventilation; NEWS2= National Early Warning Score 2; NIV= Non-Invasive Ventilation; P/F ratio= PiO2/FiO2 ratio.
